# Adiponectin levels in maternal serum and umbilical cord blood at birth by mode of delivery: relationship to anthropometric measurements and fetal sex

**DOI:** 10.1186/s12884-019-2460-y

**Published:** 2019-10-07

**Authors:** Seyedeh Razieh Fazeli Daryasari, Najmeh Tehranian, Anoshirvan Kazemnejad, Fatemeh Razavinia, Fatemeh Tork Tatari, Fattaneh Pahlavan

**Affiliations:** 1 Department of Midwifery & Reproductive Health, Faculty of Medical Sciences, Tarbiat Modares University, Jalal Ale Ahmad Highway, P.O.Box: 14115-111, Tehran, Iran; 2 Department of Biostatistics, Faculty of Medical Sciences, Tarbiat Modares University, Tehran, Iran

**Keywords:** Adiponectin, Maternal serum, Umbilical cord blood, Mode of delivery

## Abstract

**Background:**

The rate of cesarean section is increasing worldwide. Adiponectin is a hormone related to anti-inflammatory and anti-atherogenic effects; and it′s concentrations may change in response to inflammatory situations including surgical intervention. The aim of the current study was to investigate serum adiponectin levels in maternal and umbilical cord blood according to different modes of delivery and their relationship with anthropometric measurements and fetal sex.

**Methods:**

The study population initially comprised 90 healthy pregnant women referred to the teaching hospital. Eventually, 40 participants in the vaginal delivery group and 35 subjects in the cesarean delivery group were recruited in to the study. Umbilical cord blood and maternal serum samples were analyzed according to the standard protocol from the manufacturer. The collected data were analyzed using SPSS-16 software. *P*-value < 0.05 was considered as the significance level for all tests.

**Results:**

Our results indicated a significant association between maternal adiponectin and the mode of delivery, with adiponectin levels significantly higher in vaginal delivery compared to cesarean section (*P* < 0.001). However, no difference was found in umbilical cord blood adiponectin between the two groups (*P* = 0.51). A significant positive correlation was found between maternal serum adiponectin in the first day after birth and umbilical cord blood adiponectin in the vaginal delivery group (*P* = 0.007). Nevertheless, this correlation was not statistically significant in the cesarean delivery group (*P* = 0.62). There was also no significant correlation between fetal sex and anthropometric measurements with maternal adiponectin (*P* = 0.44) and umbilical cord blood adiponectin (*P* = 0.86).

**Conclusions:**

The result of the current study revealed that maternal adiponectin concentration was significantly higher in vaginal delivery compared to cesarean section, which might be due to the increased levels of maternal adiponectin release during labor.

## Background

Adiponectin as an active polypeptide hormone belongs to the adipokine family, which is secreted mainly from adipose tissue [1, 2] and increases insulin sensitivity [3] with anti-inflammatory and anti-atherogenic effects [4, 5]. Hypoadiponectinemia as an inflammatory marker can cause metabolic syndrome [6] and a higher incidence of diabetes [7]. Furthermore, low adiponectin levels are related to cardiovascular diseases, high blood pressure and obesity, especially visceral obesity [8–10]. Adiponectin concentrations and insulin sensitivity tend to decrease progressively in the first trimester of pregnancy [11–13]. These changes play a potential role in the enhancement of gluconeogenesis and lipolysis, and the reduction of glucose consumption in maternal muscle and fat tissue [14, 15]. In addition, it can be assumed that high production of adiponectin in the placenta and the fetus are associated with fetal growth [16].

In recent years, the rate of cesarean section (C-section) deliveries is suggestive of an increasing trend worldwide [17]. C-section as a surgical procedure is usually extremely stressful experience which can lead to an inflammatory situation and change the concentration of adiponectin [18]. The results of a systematic review revealed that C-section is associated with an increased risk of type-1 diabetes, asthma and metabolic syndrome as long-term outcomes [19].

We hypothesized that adiponectin concentrations would differ with respect to modes of delivery. Considering the rapidly rising C-section delivery rate worldwide and the little information on the role of adiponectin in fetal development; our study aimed to investigate the difference between serum adiponectin levels in maternal and umbilical cord blood according to different modes of delivery in Iranian women.

## Methods

### Study population

In this prospective cohort study, the study population initially comprised 90 healthy pregnant women referred to the teaching hospitals of Mahdieh Tehran and Kamali in Karaj between May and June 2016. The sample size was calculated based on the following formula and the study of Hermansson et al. [20] with 95% confidence interval and 95% power of test which equals to 33 women in each group and by considering the probability of withdrawal rate (about 30%); 45 samples were eventually included in each group of the study. Five women in the normal delivery group and ten women in the elective cesarean section group failed to meet our eligibility criteria. Finally, 40 participants in the vaginal delivery group and 35 subjects in the cesarean delivery group were recruited in to the study.

$$ \mathrm{N}={\left({\mathrm{Z}}_{1\hbox{-} \upalpha /2}+{\mathrm{Z}}_{1\hbox{-} \mathrm{B}}\right)}^2\times \left({{\mathrm{S}}_1}^2+{{\mathrm{S}}_2}^2\right)\div {\left({\overline{\mathrm{X}}}_1\hbox{-} {\overline{\mathrm{X}}}_2\right)}^2 $$

Z_1-α/2_ = 1.96

Z_1-B_ = 1.64

(S_1_) Standard deviation of serum adiponectin after normal delivery = 7.3

(S_2_) Standard deviation of serum adiponectin after elective cesarean section = 6.8

$\left({\overline{\mathrm{X}}}_1\right)$ The mean serum adiponectin levels after normal delivery= 21.6

$\left({\overline{\mathrm{X}}}_2\right)$ The mean serum adiponectin levels after elective cesarean section =15.3

$$ \mathrm{n}=\left[{\left(1.96+1.64\right)}^2\ \left({7.3}^2+{6.8}^2\right)\right]\div {\left(21.6-15.3\right)}^2=32.6\approx 33 $$

The eligibility criteria included age between 18 and 40 years, gestational age ≥ 37 weeks, singleton pregnancy, the absence of any psychiatric disorders, and the lack of any systemic and chronic diseases such as Lupus, fatty liver, and thyroid disorders. Participants were excluded in the event of tobacco and/or alcohol consumption, any history of gastric surgery or gastric ulcer disease, gestational diabetes, preeclampsia; experiencing unusual tensions during pregnancy such as divorce, assisted vaginal delivery such as using forceps and / or vacuums, and cesarean section with hysterectomy.

### Anthropometry

The gestational age was calculated from the first day of the last menstrual period (LMP); however, if there was still any uncertainty about the data, the first ultrasound within the first trimester of pregnancy was performed. Maternal weight and height were measured; and body mass index (BMI: weight [kg]/height^2^ [m]) was calculated using the standard equation and single investigator. The infants’ weight, length, and the circumference of the head and chest were measured at birth.

### Blood collection & adiponectin measurement

Umbilical cord blood and maternal serum samples were collected without fasting after birth and within 24 h after delivery, respectively. Blood samples were immediately centrifuged at 4000 rpm at 4 °C for 10 min and aliquots of serum were frozen without delay at − 70 °C until analysis. The concentration of adiponectin (μg/ml) was measured with enzyme-linked immunosorbent assay (ELISA) using commercial kit (Human Adiponectin, Zell Bio GmbH, Ulm, Germany) according to the standard protocol from the manufacturer with a sensitivity of 0.1 μg / ml. The intra-assay coefficient of variation (CV) was 6.3%. A standardized checklist was utilized to gather anthropometric and laboratory data from mothers and infants.

### Statistical analysis

Data are presented as mean (SD). A Mann-Whitney test was used to compare serum adiponectin levels in maternal and umbilical cord blood in different modes of delivery; and to determine the relationship between maternal adiponectin and umbilical cord blood with the baby’s gender. Spearman correlation test was also utilized to determine the correlation between serum adiponectin levels in maternal and umbilical cord blood with anthropometric indices. *P*-value < 0.05 was considered as the significance level for all tests. The data were analyzed using SPSS (version 16, SPSS, Inc., Chicago, IL, USA) software.

## Results

Both groups were matched according to the demographic variables including age, maternal education, number of pregnancies, number of deliveries, number of live births, miscarriages, newborn’s sex, weight, height, head and chest circumference, and maternal BMI. We found no difference between-groups with respect to different variables (Table 1). Characteristics of the participants and their infants are presented in Table 1.

**Table 1:** Characteristics of mothers and their infants (*n* = 75)

Variables	Vaginal delivery (*n* = 40)	Cesarean section (*n* = 35)	*P*-value
Birth weight (g)	3247.37 ± 407.65^a^	3219.28 ± 382.22^a^	0.76^b^
Birth length (cm)	50.37 ± 2.09^a^	50.85 ± 2.54^a^	0.23^c^
Birth head circumference (cm)	34.83 ± 1.21^a^	34.84 ± 0.91^a^	0.83^c^
Birth chest circumference (cm)	33.71 ± 1.09^a^	33.67 ± 1.48^a^	0.72^c^
Gender			
Male	21 (52.5) ^d^	18 (51.4) ^d^	0.92^e^
Female	19 (47.5) ^d^	17 (48.6) ^d^	
Maternal age (y)			
≤ 25	14 (35) ^d^	8 (22.9) ^d^	0.26^e^
26–35	24 (60) ^d^	22 (62.9) ^d^	
> 35	2 (5) ^d^	5 (14.3) ^d^	
Maternal education			
< 12 years education	21 (52.5) ^d^	17(48.6) ^d^	0.73^e^
≥ 12 years education	19 (47.5) ^d^	18 (51.4) ^d^	
Number of live births			
0	18(45) ^d^	8 (22.9) ^d^	0.14^e^
1	16 (40) ^d^	16 (45.7) ^d^	
2	4 (10) ^d^	9 (25.7) ^d^	
≥3	2 (5) ^d^	2 (5.7) ^d^	
Number of deliveries			
0	18 (45) ^d^	8 (22.9) ^d^	0.1^e^
1	16 (40) ^d^	15 (42.9) ^d^	
2	4 (10) ^d^	10 (28.6) ^d^	
≥3	2 (5) ^d^	2 (5.7) ^d^	
Number of pregnancies			
1	16 (40) ^d^	7 (20) ^d^	0.068^e^
2	14 (35) ^d^	9 (25.7) ^d^	
3	7 (17.5) ^d^	14 (40) ^d^	
4≥	3 (7.5) ^d^	5 (14.3) ^d^	
Number of miscarriages			
0	34 (85) ^d^	24 (68.6) ^d^	0.21^e^
1	5 (12.5) ^d^	10 (28.6) ^d^	
2≥	1 (2.5) ^d^	1 (2.9) ^d^	
Maternal BMI at birth (kg/m^2^)			
18.5–24.9	13 (32.5) ^d^	5 (14.3) ^d^	0.16^e^
25–29.9	16 (40) ^d^	16 (45.7) ^d^	
30≥	11 (27.5) ^d^	14 (40) ^d^	

^a^, mean ± SD; ^b^, T-test; ^c^, Mann-Whitney; ^d^, Number (%); ^e^, Chi- square

The mean (SD) age of the study population was 28.45 (5.83) years. Almost half of the participants (49.3%) had more than 12 years of educational experience. The mean (SD) gestational age at delivery was 39.70 (+/− 0.91) weeks and 38.44 (+/− 0.37) weeks in the normal and C-section delivery groups, respectively. The result of our study indicated a significant association between maternal adiponectin and the mode of delivery with significantly higher rate in vaginal delivery compared to C-section (*P* < 0.001). However, no difference was seen in the umbilical cord blood adiponectin between the two groups (*P* = 0.51) (Table 2). According to the Spearman’s correlation, a significant positive relationship was found between maternal serum adiponectin in the first 24 h after birth and umbilical cord blood adiponectin in the vaginal delivery group (r = 0.42, *P* = 0.007); However, this correlation was not statistically significant in the cesarean delivery group (r = 0.085, *P* = 0.62) (Table 3). The infants’ mean (SD) weight, length, head and chest circumference at birth were 3234.26 ± 393.58 g, 50.60 ± 2.31 cm, 34.84 ± 1.07 cm, and 33.69 ± 1.28 cm, respectively. The concentration of adiponectin in maternal serum and umbilical cord blood based on infant’s sex are shown in Table 4. Nevertheless, no significant correlation was found between different variables and adiponectin concentration (Tables 4, 5).

**Table 2:** Comparison of serum adiponectin levels in maternal and umbilical cord blood according to different modes of delivery (*n* = 75)

Serum adiponectin levels (μg/ml)	Vaginal delivery (*n* = 40)	Cesarean section (*n* = 35)	*P*-value
Maternal adiponectin (μg/ml)	8.52 ± 3.70^a^	6.46 ± 2.45^a^	< 0.001^b^
Umbilical cord blood adiponectin (μg/ml)	8.33 ± 4.72^a^	7.33 ± 3.12^a^	0.51^b^

^a^, mean ± SD; ^b^, Mann-Whitney

**Table 3:** Correlation between maternal serum adiponectin levels in the first 24 h after birth and umbilical cord blood adiponectin (*n* = 75)

Mode of delivery	Serum adiponectin levels (μg/ml)	r	P
Vaginal delivery (*n* = 40)	Maternal adiponectin	0.42	0.007^a^
	Umbilical cord blood adiponectin		
Cesarean section(*n* = 35)	Maternal adiponectin	0.085	0.62^a^
	Umbilical cord blood adiponectin		

^a^, Spearman correlation

**Table 4:** Association between adiponectin concentrations in maternal serum & umbilical cord blood and infant’s sex (*n* = 75)

Gender	Maternal adiponectin (ug/mL)		Umbilical cord blood adiponectin (ug/mL)	
	Mean ± SD	*P*-value	Mean ± SD	*P*-value
Male (*n* = 39)	7.64 ± 3.50	0.44^a^	7.73 ± 3.68	0.86^a^
Female (*n* = 36)	7.48 ± 3.16		8.01 ± 4.48	

^a^, Mann-Whitney test

**Table 5:** Correlation between adiponectin concentrations in maternal serum & umbilical cord blood adiponectin and anthropometric measurements at birth (*n* = 75)

Anthropometric measurements	Maternal adiponectin		Umbilical cord blood adiponectin	
	r	p	r	p
Birth weight (g)	0.18	0.11^a^	0.08	0.48^a^
Birth length (cm)	0.003	0.98^a^	0.11	0.32^a^
Birth head circumference (cm)	0.18	0.12^a^	0.20	0.08^a^
Birth chest circumference (cm)	0.17	0.13^a^	0.08	0.49^a^

^a^Spearman correlation

## Discussion

To the best of our knowledge, this paper is the first study conducted in Iran to evaluate the correlation between mode of delivery and adiponectin concentration. According to our knowledge, the number of studies conducted at the international level was quiet few, especially in making comparison and correlation between cord blood and maternal adiponectin levels. The result of the current study revealed that maternal adiponectin concentration was significantly higher in vaginal delivery compared to C-section. Nevertheless, the mode of delivery did not have statistical effects on umbilical cord blood adiponectin concentrations. A similar result was obtained in Brazil, where the adiponectin concentrations were reported higher in the vaginal delivery group [18].

A plausible explanation could be the increase of corticotropin-releasing hormone (CRH) secretion, highly secreted under stressful conditions and enhances the release of the adrenocorticotropic hormone (ACTH) from the pituitary and the secretion of glucocorticoids from the adrenal gland. In addition, labor is also considered as a stressful situation. Some studies reported intraventricular injection of adiponectin would increases plasma concentrations of ACTH and CRH mRNA in the parvocellular nucleus. Moreover, adiponectin depolarizes CRH secretion neurons in vitro [21, 22].

Differences in adiponectin concentration based on the mode of delivery can also be due to the active contraction in vaginal delivery. Adiponectin affects vascular smooth muscle contraction. Noble et al. demonstrated that adiponectin enhances calcium dependency of mouse bladder contraction mediated by protein kinase Cα expression. In this study, mice with high levels of adiponectin indicated higher concentrations of intracellular Ca^2 +^_,_ and more contraction sensitivity to intracellular Ca^2+^ [23]. Although various factors can affect maternal adiponectin concentration, we couldn’t find a reasonable explanation on the correlation between mode of delivery and umbilical cord blood adiponectin. However, in the current study, this relationship was not deemed significant. In a similar study, Logan and colleagues reported a non-significant relationship between mode of delivery and umbilical cord blood adiponectin [24]. Similarly, another study reported no significant association between umbilical cord blood adiponectin and mode of delivery [25]. In contrast, Hermansson et al. reported that adiponectin concentration in umbilical cord blood was significantly higher in vaginal delivery than elective C-section [20]. This inconsistency might be due to the small number of studies on this issue.

According to the result of the present study, maternal adiponectin was positively and significantly correlated with umbilical cord blood adiponectin in vaginal delivery. Nevertheless, this correlation in C-section was not deemed significant. Similarly, some studies reported no significant association between maternal and umbilical cord blood adiponectin [26–29]. On the contrary, Luo and colleagues found a correlation between two variables [25]. A plausible explanation could be a low sample size. Compounds with the molecular weight of 500 Da can cross the placenta, whereas the molecular weight of adiponectin is 30 kilodalton which makes it impossible to cross the placenta [29]. However, due to the presence of adiponectin receptor in rat and human placenta; human placenta might be the source of adiponectin [30, 31].

Furthermore, the result of another study indicated during the term; adiponectin is expressed in the cytotrophoblast of the placenta and is secreted in both maternal and fetal blood circulation [30]. Additionally, the maternal adiponectin concentration decreases after delivery, which can indicate that the placenta plays an important role in the production of maternal adiponectin [14]. The relationship between maternal and umbilical cord blood adiponectin in the present study was not significant; this may be due to the reduced levels of adiponectin after elective C-section.

The results of this study found no significant relationship between maternal and umbilical cord blood adiponectin levels and neonate sex. In concordance with our study, Zare and colleagues reported a non-significant relationship between adiponectin concentration and gender of neonates [32]. Similarly, some studies reported no significant association between adiponectin levels and gender [26, 33–35].

In our study, no correlation was found between adiponectin concentrations and anthropometric measurements. In some similar studies, no relationship was seen between adiponectin and anthropometric measurements [29, 32, 33]. On the contrary, in a prospective study in China, multiple linear regression analysis indicated a positive correlation between umbilical cord blood adiponectin and anthropometric measurements [36]. Similarly, Weyermann et al. of all included variables, found a positive correlation only between birth weight and umbilical cord blood adiponectin [26]. This inconsistency might be due to the small number of studies; hence further clinical trial studies are needed.

One of the advantages of the current study was the evaluation of adiponectin serum levels of both mother’s and umbilical cord blood, which can effectively assess the mode of delivery and its outcome on the mother and baby simultaneously.

One limitation that could be considered for this study was that the elective cesarean delivery was not separated from emergency cesarean delivery.

## Conclusions

The result of the current study revealed that maternal adiponectin concentration was significantly lower in C-section compared to vaginal delivery. According to the inverse association between adiponectin and insulin resistance, and considering diseases such as type 2 diabetes, cardiovascular disease, high blood pressure, metabolic syndrome, and dyslipidemia; it can be assumed that the decrease in adiponectin levels in C-section delivery may increase the risk of the mentioned diseases later in the life of mothers and their neonates. Therefore, it is necessary to provide more attention to these women and to ensure that they are encouraged to have vaginal delivery.

## Data Availability

Data supporting out findings can be sent upon request.

## Abbreviations

ACTH: Adrenocorticotropic hormone
BMI: Body mass index
CRH: Corticotropin-releasing hormone
C-Section: Cesarean section
CV: Coefficient of variation
ELISA: Enzyme-linked immunosorbent assay
LMP: Last menstrual period
